# Impact of flash glucose monitoring on hypoglycaemia in adults with type 1 diabetes managed with multiple daily injection therapy: a pre-specified subgroup analysis of the IMPACT randomised controlled trial

**DOI:** 10.1007/s00125-017-4527-5

**Published:** 2017-12-23

**Authors:** Per Oskarsson, Ramiro Antuna, Petronella Geelhoed-Duijvestijn, Jens Krӧger, Raimund Weitgasser, Jan Bolinder

**Affiliations:** 1 Department of Medicine, Karolinska University Hospital Huddinge, Karolinska Institute, 141 86 Stockholm, Sweden; 2 Department of Medicine, Clinica Diabetologica, Gijon, Spain; 3 Department of Internal Medicine, Haaglanden Medisch Centrum, Den Haag, the Netherlands; 4 Department of Diabetes, Zentrum für Diabetologie Hamburg Bergedorf, Hamburg, Germany; 5 Department of Medicine, Wehrle-Diakonissen Hospital, Salzburg, Austria; 6 1st Department of Medicine, University Hospital of Paracelsus Medical Private University, Salzburg, Austria

**Keywords:** Clinical diabetes, Devices, Hypoglycaemia, Insulin therapy

## Abstract

**Aims/hypothesis:**

Evidence for the effectiveness of interstitial glucose monitoring in individuals with type 1 diabetes using multiple daily injection (MDI) therapy is limited. In this pre-specified subgroup analysis of the Novel Glucose-Sensing Technology and Hypoglycemia in Type 1 Diabetes: a Multicentre, Non-masked, Randomised Controlled Trial’ (IMPACT), we assessed the impact of flash glucose technology on hypoglycaemia compared with capillary glucose monitoring.

**Methods:**

This multicentre, prospective, non-masked, RCT enrolled adults from 23 European diabetes centres. Individuals were eligible to participate if they had well-controlled type 1 diabetes (diagnosed for ≥5 years), HbA_1c_ ≤ 58 mmol/mol [7.5%], were using MDI therapy and on their current insulin regimen for ≥3 months, reported self-monitoring of blood glucose on a regular basis (equivalent to ≥3 times/day) for ≥2 months and were deemed technically capable of using flash glucose technology. Individuals were excluded if they were diagnosed with hypoglycaemia unawareness, had diabetic ketoacidosis or myocardial infarction in the preceding 6 months, had a known allergy to medical-grade adhesives, used continuous glucose monitoring (CGM) within the previous 4 months or were currently using CGM or sensor-augmented pump therapy, were pregnant or planning pregnancy or were receiving steroid therapy for any disorders. Following 2 weeks of blinded (to participants and investigator) sensor wear by all participants, participants with sensor data for more than 50% of the blinded wear period (or ≥650 individual sensor results) were randomly assigned, in a 1:1 ratio by a central interactive web response system (IWRS) using the biased-coin minimisation method, to flash sensor-based glucose monitoring (intervention group) or self-monitoring of capillary blood glucose (control group). The control group had two further 14 day blinded sensor-wear periods at the 3 and 6 month time points. Participants, investigators and staff were not masked to group allocation. The primary outcome was the change in time in hypoglycaemia (<3.9 mmol/l) between baseline and 6 months in the full analysis set.

**Results:**

Between 4 September 2014 and 12 February 2015, 167 participants using MDI were enrolled. After screening and the baseline phase, participants were randomised to intervention (*n* = 82) and control groups (*n* = 81). One woman from each group was excluded owing to pregnancy; the full analysis set included 161 randomised participants. At 6 months, mean time in hypoglycaemia was reduced by 46.0%, from 3.44 h/day to 1.86 h/day in the intervention group (baseline adjusted mean change, −1.65 h/day), and from 3.73 h/day to 3.66 h/day in the control group (baseline adjusted mean change, 0.00 h/day), with a between-group difference of −1.65 (95% CI −2.21, −1.09; *p* < 0.0001). For participants in the intervention group, the mean ± SD daily sensor scanning frequency was 14.7 ± 10.7 (median 12.3) and the mean number of self-monitored blood glucose tests performed per day reduced from 5.5 ± 2.0 (median 5.4) at baseline to 0.5 ± 1.0 (median 0.1). The baseline frequency of self-monitored blood glucose tests by control participants was maintained (from 5.6 ± 1.9 [median 5.2] to 5.5 ± 2.6 [median 5.1] per day). Treatment satisfaction and perception of hypo/hyperglycaemia were improved compared with control. No device-related hypoglycaemia or safety-related issues were reported. Nine serious adverse events were reported for eight participants (four in each group), none related to the device. Eight adverse events for six of the participants in the intervention group were also reported, which were related to sensor insertion/wear; four of these participants withdrew because of the adverse event.

**Conclusions/interpretation:**

Use of flash glucose technology in type 1 diabetes controlled with MDI therapy significantly reduced time in hypoglycaemia without deterioration of HbA_1c_, and improved treatment satisfaction.

**Trial registration:**

ClinicalTrials.gov NCT02232698

**Funding:**

Abbott Diabetes Care, Witney, UK

**Electronic supplementary material:**

The online version of this article (10.1007/s00125-017-4527-5) contains peer-reviewed but unedited supplementary material, which is available to authorised users.


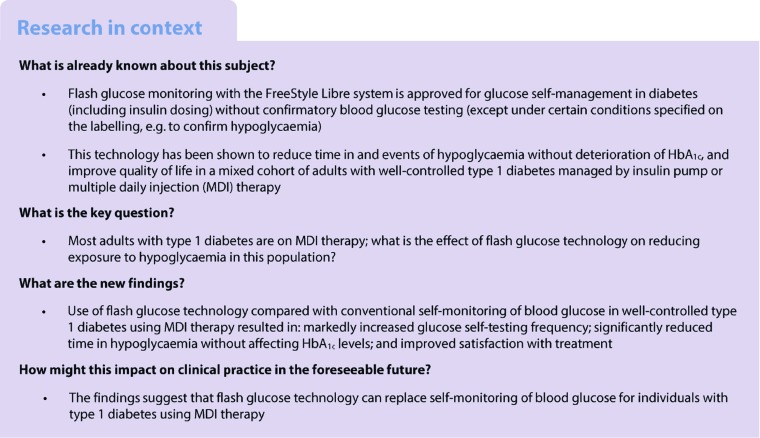


## Introduction

The benefit of optimal glucose control to delay the onset and progression of microvascular and macrovascular complications in type 1 diabetes is well established [1, 2]. The subsequent pursuit of attaining glycaemic targets with intensive insulin regimens requiring multiple daily injection (MDI) therapy or continuous subcutaneous insulin infusion (CSII) continues to be hindered by hypoglycaemia at all levels of glycaemic control (i.e. HbA_1c_) [3], thus exposing the individual to an associated and increased risk of severe hypoglycaemia [4]. Improved glucose control and/or reduced exposure to hypoglycaemia using conventional continuous glucose monitoring (CGM) technologies has been demonstrated in well-controlled [5, 6] and suboptimally controlled type 1 diabetes [7–10]. While most individuals with type 1 diabetes are on MDI therapy, these studies have been restricted to participants using CSII only [10], or in mixed cohorts of CSII and MDI users [5–9], and in both adult and paediatric participants [5–8, 10]. Accordingly, the benefit of CGM in combination with MDI therapy only has been a matter of uncertainty. Recently published data, however, showed improvements in glucose control, and secondary outcome analysis indicated reduced time in hypoglycaemia with the use of CGM in adults with type 1 diabetes on MDI therapy [11, 12].

In contrast to earlier studies, the recent Novel Glucose-Sensing Technology and Hypoglycemia in Type 1 Diabetes: a Multicentre, Non-masked, Randomised Controlled Trial (IMPACT) was specifically designed to investigate use of a novel sensor-based flash glucose monitoring system for reducing hypoglycaemia compared with conventional self-monitoring of blood glucose (SMBG) in adults with well-controlled type 1 diabetes [13]. Unlike CGM, the FreeStyle Libre system (Abbott Diabetes Care, Witney, UK) is factory calibrated and needs no calibration against SMBG during the 14 day wear time. Glucose data are transferred from the sensor to a reader when the user actively scans the sensor; otherwise the measurements are automatically captured and stored on the sensor and displayed on the reader when scanned. In the trial, one-third of the participants were CSII users and two-thirds were managed with MDI. Here, we present the results of a pre-specified subgroup analysis in which we assessed the effect of flash glucose monitoring on hypoglycaemia in the participants using MDI.

## Methods

**Fig. 1:**
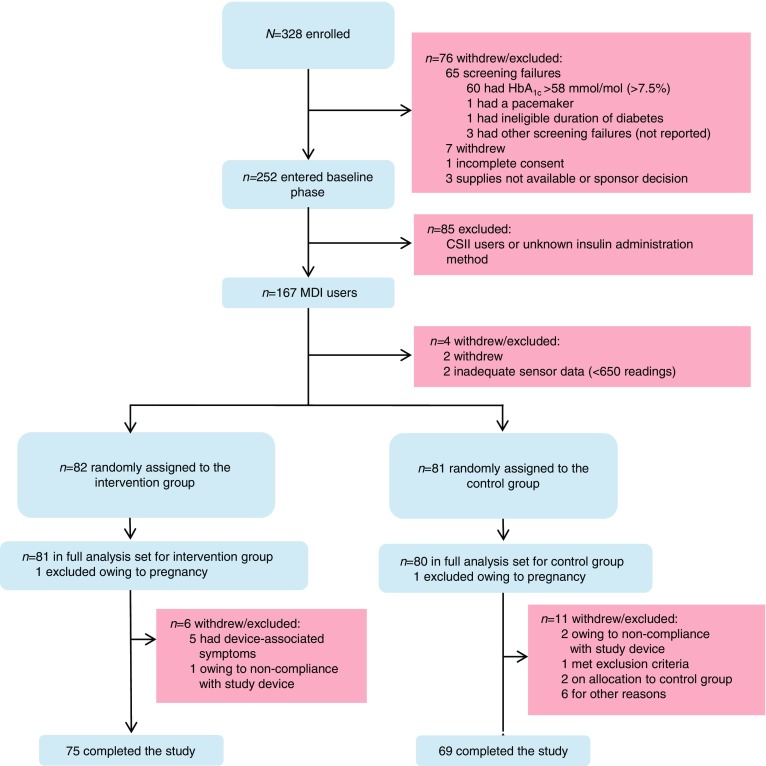
Trial profile

We used FreeStyle Libre, a sensor-based flash glucose monitoring system. The detailed rationale, methods and results of IMPACT have been described previously [13]. Briefly, this was a 6 month, multicentre, prospective, non-masked, randomised controlled trial conducted at 23 European diabetes centres (three in Sweden, six in Austria, five in Germany, three in Spain and six in the Netherlands). Of 328 participants enrolled, 252 entered the baseline phase, including 167 MDI users (Fig. 1). The aim of the study was to assess the efficacy of flash glucose monitoring technology [14] compared with conventional SMBG in adult participants with well-controlled type 1 diabetes.

At each study centre, any potentially eligible individual from the general diabetes population was invited to participate in the study if they were aged 18 years or older, had been diagnosed with type 1 diabetes for 5 years or longer, had been on their current insulin regimen for at least 3 months with an HbA_1c_ level of 58 mmol/mol (7.5%) or lower, reported SMBG on a regular basis (equivalent to ≥3 times/day) for 2 months or more and were considered by the investigator to be technically capable of using the flash sensor-based glucose monitoring system.

Individuals were not included if they were diagnosed with hypoglycaemia unawareness, had diabetic ketoacidosis or myocardial infarction in the preceding 6 months, had a known allergy to medical-grade adhesives, used CGM within the previous 4 months or were currently using CGM or sensor-augmented pump therapy, were pregnant or planning pregnancy or were receiving steroid therapy for any disorders.

Approval was given by the appropriate competent authority in each country. All participating centres gave ethical approval before the study and all participants gave written informed consent. Original data are stored at each study centre.

Following 2 weeks of blinded (to participants and investigators) sensor wear, participants with sensor data for more than 50% of the blinded wear period (or ≥650 individual sensor results) were randomly assigned in a 1:1 ratio, by a central interactive web response system (IWRS) using the biased-coin minimisation method (study centre and type of insulin administration were prognostic factors), to flash sensor-based glucose monitoring (intervention group) or SMBG (control group). Participants, investigators and staff were not masked to group allocation.

For the 6 month treatment phase (post-randomisation), control participants continued with the use of SMBG concentrations (FreeStyle Lite, Abbott Diabetes Care) to support self-management of glucose levels. This group had two further 14 day blinded sensor-wear periods before the 3 and 6 month time points. For participants in the intervention group, the sensor-based glucose monitoring system [14] was unblinded, allowing for continuous use of the sensor glucose data for self-management of glucose levels, including insulin dose decisions, in accordance with the product labelling. No training was provided for these participants for interpretation of glucose sensor data. Their historical data were uploaded at subsequent study visits, and glucose reports (including ambulatory profile reports [AGPs]) were generated for review by the healthcare professional with the participant, using the device software [15]. Intervention participants were not provided with specific training on how to use the glucose reports and neither standardised insulin titration algorithms or treatment protocols were used in the trial.

Detailed outcomes for IMPACT have been described [13]. The primary effectiveness endpoint was the difference in time spent in hypoglycaemia (<3.9 mmol/l) for the 14 days preceding the end of the 6 month study period (days 194–208) between intervention and control groups. Pre-specified secondary endpoints were sensor-derived glycaemic measures at days 194–208, day 208 HbA_1c_, change in total daily dose of insulin from day 1 to day 208, system utilisation for days 15–208 (defined as the percentage of data collected, relative to continuous device wear) and frequency of glucose finger-sticks and sensor scans per day during the study period. Sensor-derived glycaemic measures comprised: (1) number and duration of hypoglycaemic events (measured as sensor glucose <3.9 mmol/l in 24 h, by day [06:00–23:00 hours] and night [23:00–06:00 hours], or sensor glucose <3.1 or <2.2 mmol/l in 24 h); (2) time with glucose in the range of 3.9 to 10.0 mmol/l; (3) number and duration of hyperglycaemic events (>10.0 mmol/l and >13.3 mmol/l); and (4) glucose variability measurements [16]. For number and duration of events, an event was defined as at least two consecutive readings, at 15 min intervals, outside the predefined glucose range. Event duration was calculated from the first reading outside the range to the first reading returning within the range. Additional outcomes assessed in the clinical study report were proportion of participants who achieved time spent in hypoglycaemia (<3.9 mmol/l) for ≤1 h/day, body weight and BMI. Questionnaire results for the user questionnaire (participant [intervention group only] and healthcare professional) were assessed at 6 months, with patient-reported outcome measures (Diabetes Distress Scale [DDS] [17], Diabetes Quality of Life Questionnaire [DQoL] [18], Diabetes Treatment Satisfaction Questionnaire [DTSQ] [19] and Hypoglycaemia Fear Survey [HFS] [20]) assessed at baseline and at 6 months. Safety endpoints incorporated all adverse events and sensor insertion-site symptoms monitored throughout the study.

### Data analysis

We assessed the primary endpoint for this pre-specified subgroup using analysis of covariance comparing treatment groups with baseline time in hypoglycaemia as a covariate (see electronic supplementary material [ESM] Table 1 for the average number of glucose readings used in the primary endpoint analysis). Missing values were imputed by last observation carried forward. This included the baseline value if no measurements after baseline were available (see ESM Table 2). Changes in patient-reported outcome measures and quality of life were calculated by comparing scores from control and intervention group participants using analysis of covariance on baseline values. CIs were calculated for the group least squares mean of each measure and the difference between group least squares means. Data analysis was performed by a contract research organisation (ICON, Dublin, Ireland), managed by Abbott Diabetes Care, and by Abbott Diabetes Care. We used SAS version 9.2 or higher for all analyses. The trial is registered ClinicalTrials.gov (registration no. NCT02232698).

## Results

We enrolled 328 participants in total between 4 September 2014 and 12 February 2015; of these, 167 MDI users entered the baseline phase, two MDI participants were withdrawn prior to randomisation owing to inadequate sensor data and 163 participants were subsequently randomly assigned to the intervention (*n* = 81) or control group (*n* = 80). One woman from each group was excluded owing to pregnancy; the full analysis set included 161 randomised participants (Fig. 1 and Table 1).

**Table 1:** Baseline characteristics

Characteristic	Intervention (*n* = 81)	Control (*n* = 80)
Male	56 (69)	47 (59)
White	81 (100)	80 (100)
Age, years	42 (32–53)	44 (34–53)
BMI, kg/m^2^	25.1 ± 3.9	25.1 ± 3.7
Duration of diabetes, years	19 (14–25)	19 (11–31)
Screening HbA_1c_, mmol/mol	50.1 ± 4.9	49.3 ± 6.9
Screening HbA_1c_, %	6.7 ± 0.5	6.7 ± 0.6
Self-reported BG monitoring frequency per day	5.2 ± 2.1	5.2 ± 2.2
Insulin (daily dose)		
Basal, units	25.7 ± 13.9	20.9 ± 10.0
Bolus, units	24.2 ± 13.5	22.2 ± 13.4

Data are *n* (%), median (interquartile range) or mean ± SD

BG, blood glucose

**Fig. 2:**
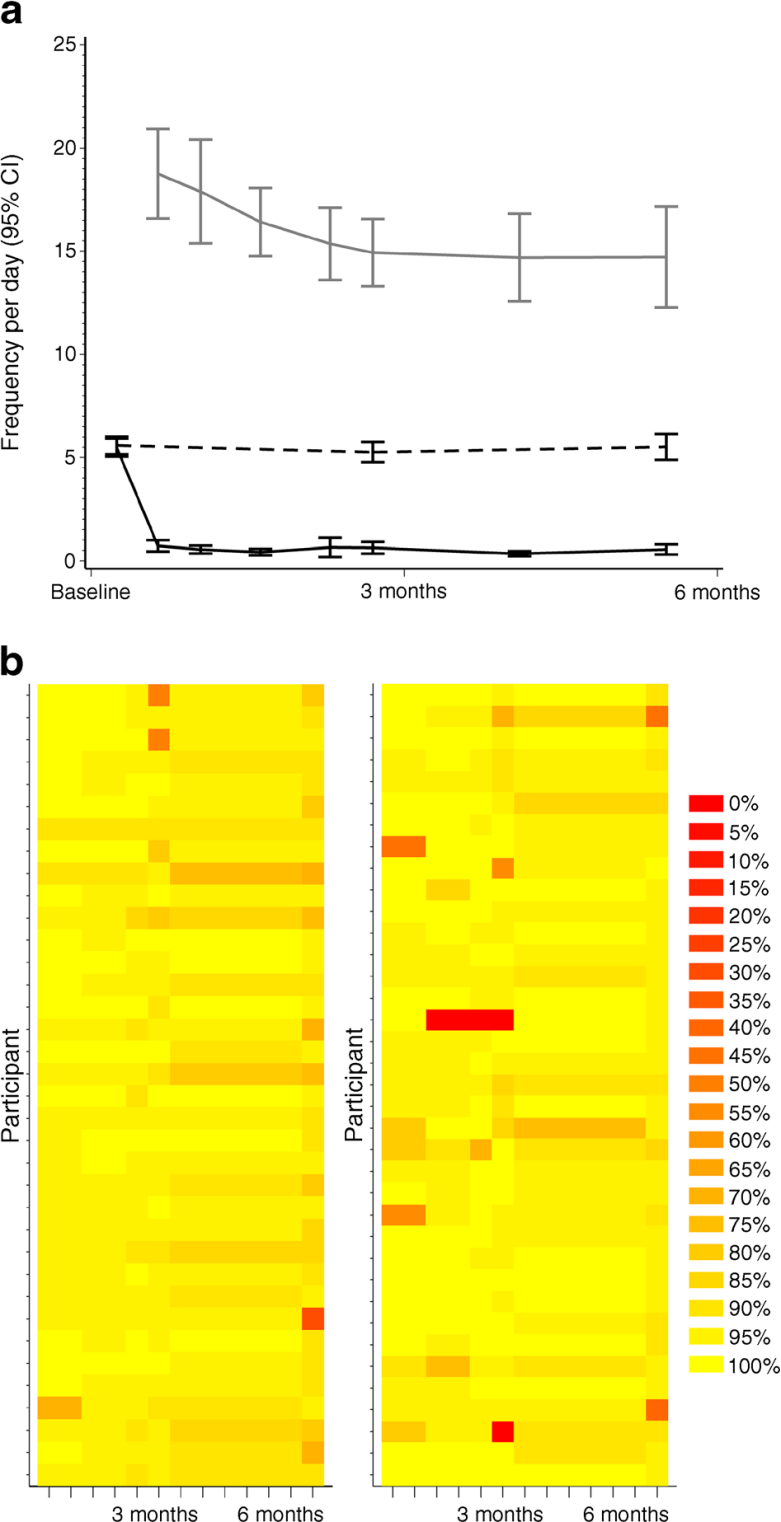
(**a**) Glucose monitoring frequency in the full analysis set. Grey line, sensor scans in intervention group; black solid line, blood glucose tests in intervention group; black dashed line, blood glucose tests in control group. (**b**) System utilisation by participant and visit intervals in the per-protocol set

The mean number of sensor scans for the intervention group was over 18 per day immediately after the device was unmasked and sensor glucose information could be used (Fig. 2a). Scanning frequency was 14.7 ± 10.7 (mean ± SD [median 12.3]) per day in the final phase (days 194–208; Fig. 2a). The median sensor-wear duration was 13.4 days (mean ± SE, 10.0 ± 0.13 days). The number of SMBG tests performed per day by intervention participants at baseline (days 1–15) was 5.5 ± 2.0 (mean ± SD) (median 5.4) falling to 0.5 ± 1.0 (median 0.1) in the final phase. Frequency of SMBG tests by control group participants was 5.6 ± 1.9 (median 5.2) per day at baseline and maintained at the final phase (5.5 ± 2.6 [median 5.1] per day) (Fig. 2a). System utilisation, defined as the percentage of data collected relative to continuous device wear for 6 months, by the intervention group (*n* = 76, including one participant that withdrew at 6 months) was 92.5 ± 8.1% (median 95.0%) (Fig. 2b).

**Fig. 3:**
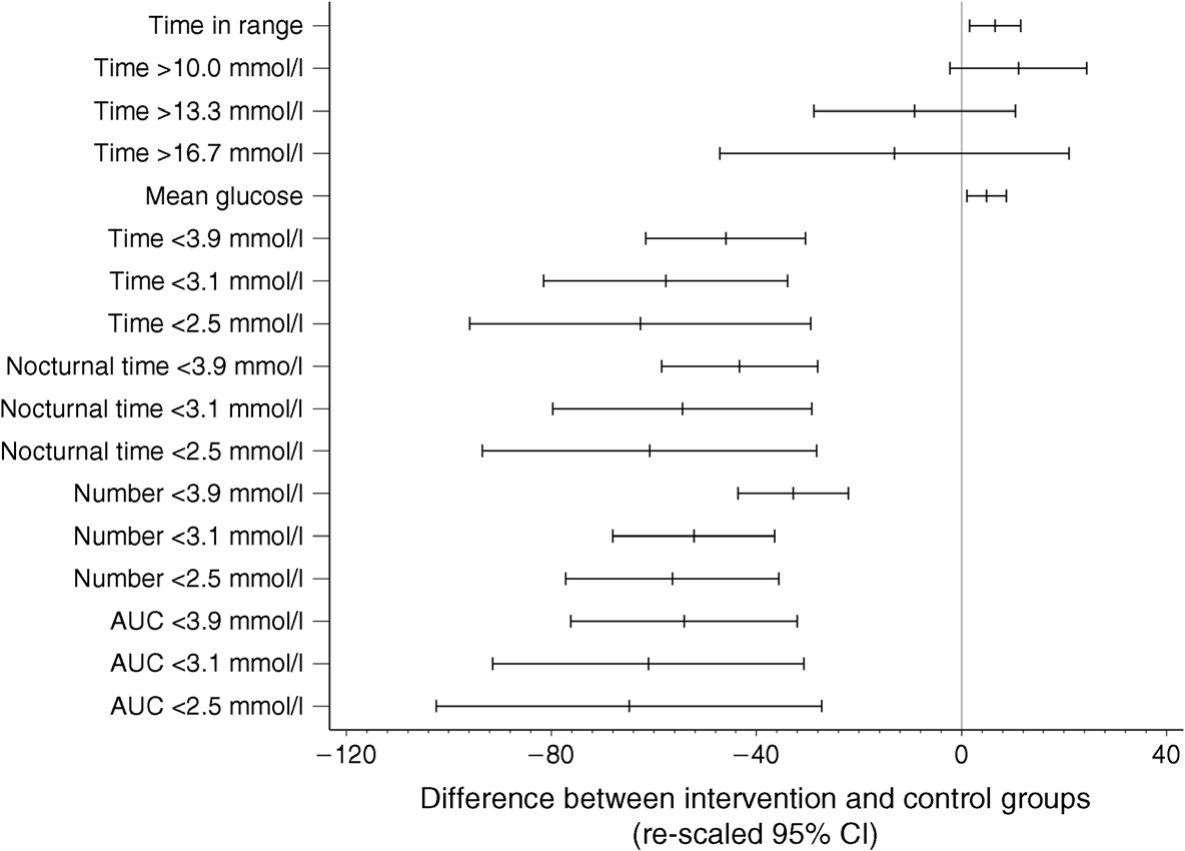
Difference in groups for change in glycaemic measures. Re-scaled 95% CIs represent the difference in the intervention group compared with the control group at 6 months, expressed as a percentage of the control group-adjusted mean

Glycaemic metrics at 6 months are given in Table 2 and Fig. 3. Time in hypoglycaemia (sensor glucose <3.9 mmol/l) reduced from 3.44 h/day to 1.86 h/day in intervention participants (baseline-adjusted mean change −1.65 h/day) and from 3.73 h/day to 3.66 h/day in the control group (baseline adjusted mean change 0.00 h/day). The adjusted between-group difference was 1.65 h/day (95% CI −2.21, −1.09 h/day), (*p* < 0.0001), a 46% reduction in time in hypoglycaemia for intervention participants compared with control. Time in hypoglycaemia at sensor glucose levels <3.1 mmol/l, <2.5 mmol/l and <2.2 mmol/l were all highly significantly reduced for the intervention group compared with control participants. The number of events below all hypoglycaemic sensor glucose levels was significantly reduced for intervention participants compared with control (Table 2 and Fig. 3). AUC was also improved for intervention compared with control participants.

**Table 2:** Glycaemic and glucose variability measures

Variable	Baseline		Study end		Difference in adjusted means between intervention and control groups (95% CI)	Difference in intervention vs control (%)	*p* value
	Intervention (*n* = 81)	Control (*n* = 79)^a^	Intervention (*n* = 81)	Control (*n* = 79)^a^			
HbA_1c_ (mmol/mol)	50.8 (4.8)	49.9 (7.5)	53.0 (6.5)	52.0 (7.5)	0.3 (−1.4, 2.0)	NA	0.77
HbA_1c_ (%)	6.80 (0.44)	6.71 (0.69)	7.00 (0.60)	6.91 (0.69)	0.02 (−0.13, 0.18)	NA	0.77
Time in glucose 3.9–10.0 mmol/l (h)	15.0 (2.6)	14.3 (2.9)	15.7 (2.8)	14.3 (3.0)	0.9 (0.2, 1.7)	6.5	0.011
Glucose <3.9 mmol/l							
24 h period							
Events	1.80 (0.80)	1.72 (0.75)	1.23 (0.69)	1.78 (0.78)	−0.59 (−0.78, −0.40)	−32.8	<0.0001
Duration (h)	3.44 (2.10)	3.73 (2.72)	1.86 (1.36)	3.66 (2.79)	−1.65 (−2.21, −1.09)	−46.0	<0.0001
AUC (h × mmol/l)	3.17 (2.57)	3.60 (3.38)	1.48 (1.49)	3.56 (3.79)	−1.87 (−2.63, −1.10)	−54.1	<0.0001
Night period (23:00–06:00 hours)							
Events	0.57 (0.34)	0.61 (0.38)	0.30 (0.26)	0.54 (0.33)	−0.22 (−0.30, −0.14)	−41.7	<0.0001
Duration (h)	1.20 (0.89)	1.41 (1.12)	0.61 (0.64)	1.28 (1.09)	−0.57 (−0.81, −0.34)	−46.6	<0.0001
Glucose <3.1 mmol/l							
24 h period							
Events	1.01 (0.65)	1.00 (0.69)	0.50 (0.48)	1.04 (0.76)	−0.55 (−0.71, −0.38)	−52.2	<0.0001
Duration (h)	1.75 (1.53)	1.99 (1.97)	0.75 (0.88)	1.97 (2.24)	−1.10 (−1.55, −0.65)	−57.7	<0.0001
AUC (h × mmol/l)	1.00 (1.07)	1.20 (1.39)	0.40 (0.58)	1.20 (1.71)	−0.71 (−1.06, −0.36)	−61.1	0.0001
Night period (23:00–06:00 hours)							
Events	0.37 (0.27)	0.41 (0.34)	0.16 (0.18)	0.33 (0.27)	−0.16 (−0.22, −0.09)	−47.6	<0.0001
Duration (h)	0.67 (0.62)	0.85 (0.85)	0.28 (0.37)	0.76 (0.86)	−0.39 (−0.57, −0.21)	−54.4	<0.0001
Glucose <2.5 mmol/l							
24 h period^b^							
Events	0.61 (0.55)	0.63 (0.59)	0.28 (0.36)	0.65 (0.66)	−0.37 (−0.50, −0.23)	−56.4	<0.0001
Duration (h)	0.97 (1.15)	1.19 (1.48)	0.38 (0.62)	1.20 (1.84)	−0.72 (−1.11, −0.34)	−62.6	0.0003
AUC (h × mmol/l)	0.26 (0.34)	0.32 (0.44)	0.10 (0.18)	0.33 (0.57)	−0.21 (−0.33, −0.09)	−64.8	0.0008
Night period (23:00–06:00 hours)^b^							
Events	0.26 (0.25)	0.30 (0.32)	0.10 (0.15)	0.23 (0.24)	−0.12 (−0.17, −0.06)	−51.3	<0.0001
Duration (h)	0.40 (0.46)	0.56 (0.69)	0.15 (0.25)	0.50 (0.73)	−0.28 (−0.44, −0.13)	−60.8	0.0003
Glucose <2.2 mmol/l							
24 h period							
Events	0.44 (0.48)	0.49 (0.52)	0.20 (0.32)	0.52 (0.63)	−0.30 (−0.43, −0.17)	−58.6	<0.0001
Duration (h)	0.69 (0.97)	0.88 (1.24)	0.27 (0.53)	0.94 (1.66)	−0.59 (−0.94, −0.24)	−65.6	0.0012
Duration (h) at hyperglycaemic glucose level within 24 h period							
>10.0 mmol/l	5.6 (2.4)	6.0 (3.3)	6.4 (3.0)	6.0 (3.3)	0.7 (−0.1, 1.4)	11.1	0.10
>13.3 mmol/l	1.77 (1.36)	2.05 (1.86)	1.78 (1.41)	2.10 (1.62)	−0.19 (−0.58, 0.21)	−9.2	0.36
>16.7 mmol/l	0.44 (0.50)	0.57 (0.77)	0.37 (0.47)	0.47 (0.57)	−0.06 (−0.21, 0.09)	−13.1	0.45
Glucose variability							
BGRI	8.1 (2.3)	8.7 (2.9)	7.4 (2.5)	8.6 (2.7)	−0.8 (−1.4, −0.1)	−9.4	0.017
CV glucose (%)	43.2 (6.6)	43.4 (6.5)	37.8 (5.6)	42.6 (6.8)	−4.7 (−6.2, −3.2)	−11.1	<0.0001
LBGI	2.70 (1.35)	2.87 (1.76)	1.61 (0.93)	2.77 (1.73)	−1.07 (−1.42, −0.72)	−39.3	<0.0001
MAGE (mmol/l)	7.9 (1.5)	8.2 (1.8)	7.5 (1.4)	8.0 (1.8)	−0.31 (−0.72, 0.11)	−3.9	0.14
Mean glucose (mmol/l)	7.8 (1.0)	7.9 (1.4)	8.2 (1.1)	7.9 (1.3)	0.38 (0.08, 0.68)	4.9	0.014
SD of glucose (mmol/l)	3.36 (0.63)	3.41 (0.76)	3.10 (0.58)	3.36 (0.78)	−0.23 (−0.39, −0.07)	−6.9	0.0051
CONGA 2 h (mmol/l)	3.2 (0.7)	3.2 (0.8)	2.8 (0.7)	3.3 (0.8)	−0.48 (−0.66, −0.30)	−14.8	<0.0001
CONGA 6 h (mmol/l)	4.0 (1.5)	4.0 (1.5)	3.7 (1.4)	4.1 (1.6)	−0.39 (−0.85, 0.06)	−9.7	0.089

Data are mean (SD) unless otherwise stated

^a^Baseline sensor data was not available for analysis for one control participant

^b^Post hoc endpoint

BGRI, blood glucose risk index; CONGA, continuous overall net glycaemic action; LBGI, low blood glucose index; MAGE, mean amplitude of glycaemic excursions

**Fig. 4:**
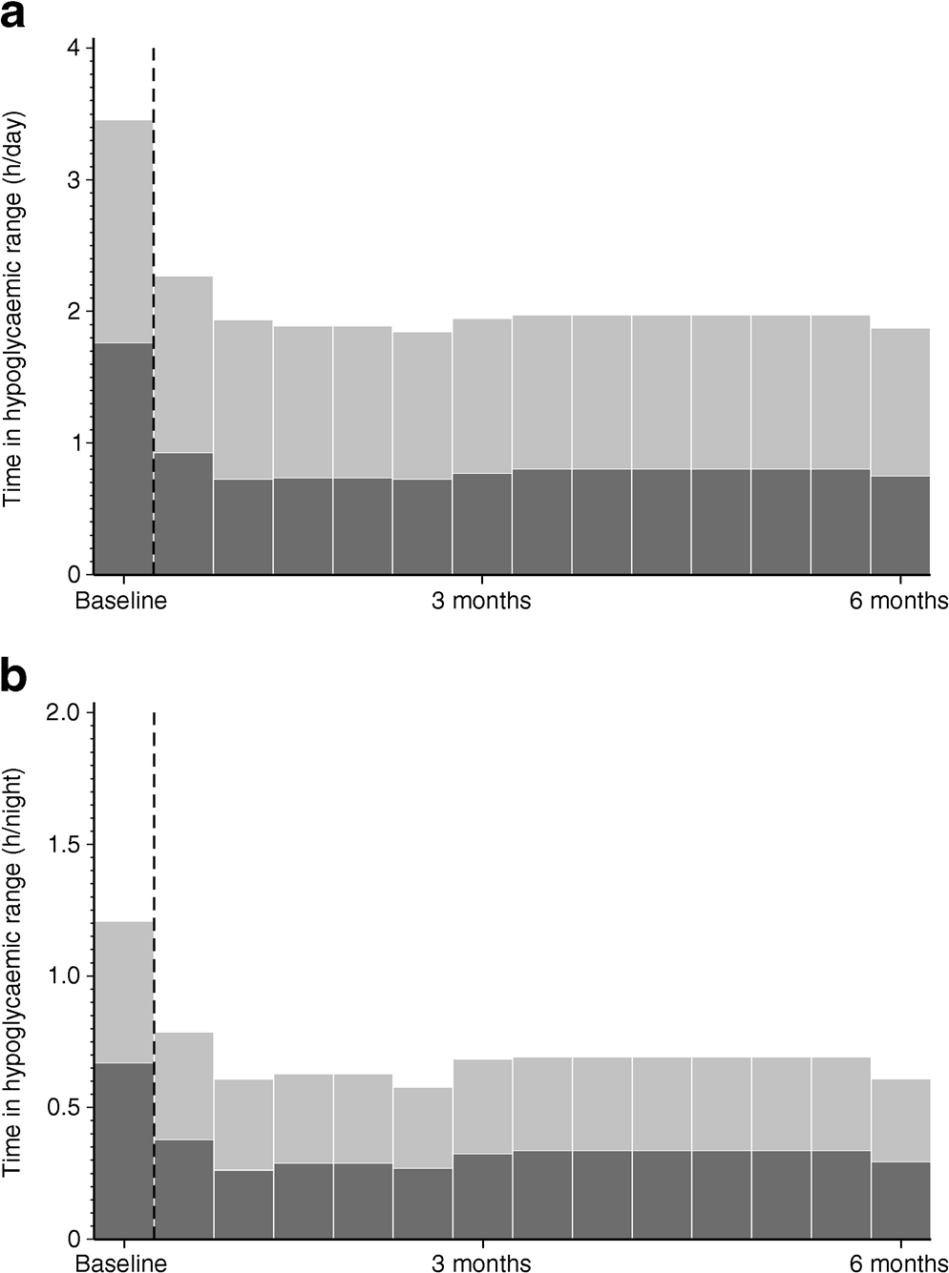
Time in hypoglycaemic range during baseline and treatment phase (days 1–208) in the intervention group in the per-protocol set for (**a**) overall 24 h and (**b**) the night (23:00–06:00 hours) period. Light grey, time in range 3.1–<3.9 mmol/l; dark grey, time <3.1 mmol/l; dashed line, sensor unblinded

Time and number of nocturnal hypoglycaemia events (23:00–06:00 hours) were reduced in favour of the intervention group compared with the control group (Table 2 and Fig. 3). In the intervention group, the reduction of time in daily and night-time hypoglycaemia was evident almost immediately after starting active use of the device (Fig. 4a, b). The intervention group had more participants at 6 months with ≤1 h/day in hypoglycaemia (<3.9 mmol/l) compared with control participants (33% vs 10%, *p* = 0.0005). Time in hyperglycaemia (>10.0 mmol/l) was comparable between the intervention and control groups (*p* = 0.10). Time in range (sensor glucose 3.9–10.0 mmol/l) was increased for intervention compared with the control group by 0.9 h/day (95% CI 0.2, 1.7 h/day, *p* = 0.011). Mean sensor glucose increased by 0.38 mmol/l (95% CI 0.08, 0.68 mmol/l), (*p* = 0.014).

At 6 months, mean HbA_1c_ was similar between the intervention and control groups (*p* = 0.77). A number of glycaemic variability measures were analysed and differences observed which favoured the intervention compared with control group, including blood glucose risk index, standard deviation of glucose, continuous overall net glycaemic action (1, 2 and 4 h), low blood glucose index and glucose coefficient of variation (Table 2). Similar data with glycaemic and glucose variability measures were also recorded after 3 months (ESM Table 3).

Over the course of the study (6 months), change in total daily dose of insulin was similar for intervention and control participants at −2.7 (SD 7.3) and −3.0 (SD 6.4) units, respectively (*p* = 0.80). There was no change in basal/bolus insulin ratio for either group.

At the end of the study, weight (*p =* 0.34) and BMI (*p =* 0.32) were comparable between the groups.

DQoL satisfaction with treatment score was improved for the intervention group compared with control participants (*p* < 0.0001), as was the DTSQ overall treatment satisfaction score (*p* < 0.0001), perception of hypoglycaemia (*p* = 0.010) and perception of hyperglycaemia scores (*p* < 0.0001 [Table 3]). Hypoglycaemia fear behaviour (*p =* 0.76) or worry (*p* = 0.59) scores and diabetes distress score (*p* = 0.98) were similar for both groups.

**Table 3:** Scores from DTSQ, DQoL, DDS and HFS questionnaires

Questionnaire item	Baseline		Study end		Difference in adjusted means in intervention and control (95% CI)	*p* value
	Intervention (*n* = 78)	Control (*n* = 70)	Intervention (*n* = 78)	Control (*n* = 70)		
DTSQ						
Total treatment satisfaction score	28.3 (4.7)	27.7 (5.3)	13.3 (5.4)	6.8 (6.2)	6.4 (4.4, 8.4)	<0.0001
Perceived frequency of hypoglycaemia	2.3 (1.2)	2.6 (1.4)	−0.4 (1.6)	0.2 (1.1)	−0.6 (−1.1, −0.2)	0.010
Perceived frequency of hyperglycaemia	2.5 (1.3)	2.8 (1.4)	−0.6 (1.7)	0.6 (1.2)	−1.2 (−1.7, −0.7)	<0.0001
DQoL						
Total core scale score	1.9 (0.3)	2.1 (0.5)	1.9 (0.4)	2.1 (0.5)	−0.1 (−0.2, 0.0)	0.15
Satisfaction with treatment	2.0 (0.5)	2.1 (0.5)	1.8 (0.5)	2.2 (0.5)	−0.3 (−0.4, −0.1)	<0.0001
Social worry	1.6 (0.5)	1.9 (0.7)	1.7 (0.6)	1.9 (0.6)	0.0 (−0.1, 0.2)	0.74
Diabetes worry	2.0 (0.6)	2.1 (0.7)	1.9 (0.6)	2.1 (0.7)	−0.1 (−0.3, 0.1)	0.23
Impact of treatment	2.0 (0.3)	2.2 (0.4)	2.0 (0.3)	2.2 (0.4)	−0.0 (−0.1, 0.1)	0.82
DDS						
Total DDS score	1.9 (1.0)	2.1 (1.1)	1.9 (1.0)	2.0 (1.0)	0.0 (−0.2, 0.2)	0.98
Emotional burden subscore	2.0 (1.0)	2.3 (1.2)	2.0 (1.1)	2.2 (1.1)	−0.0 (−0.3, 0.2)	0.77
Physician distress subscore	1.8 (1.2)	1.9 (1.3)	1.8 (1.4)	1.8 (1.2)	0.1 (−0.2, 0.4)	0.45
Regimen distress subscore	2.1 (1.1)	2.2 (1.2)	2.0 (1.1)	2.1 (1.1)	−0.0 (−0.3, 0.2)	0.71
Interpersonal distress subscore	1.6 (0.8)	2.0 (1.3)	1.6 (1.0)	1.8 (1.2)	0.0 (−0.2, 0.3)	0.74
HFS						
Behavioural subscale	11.9 (6.4)	12.7 (7.3)	13.4 (5.6)	14.2 (7.3)	−0.3 (−2.0, 1.4)	0.76
Worry subscale	15.0 (10.1)	19.0 (14.0)	14.9 (11.8)	18.4 (13.5)	−1.0 (−4.6, 2.6)	0.59

Data are mean (SD) unless otherwise stated

Participants were requested to complete questionnaires at 6 months. Hence, data from *n* = 5 participants who did not complete the study (*n* = 4 from the intervention group and *n* = 1 from the control group) are included in the analysis. Questionnaire data was not available for *n* = 1 individual in the intervention group who did complete the study

DTSQ treatment satisfaction scores range from −18 to 18; high scores indicate much more satisfied, convenient, flexible or likely to recommend treatment now. DTSQ perceived frequency scores range from −3 to 3; high scores indicate much more of the time now. DQoL scores range from 1 to 5; high scores indicate dissatisfaction, frequent impact or frequent worry. DDS scores range from 1 to 6; high scores indicate a very serious problem. HFS behaviour scores range from 0 to 40 and HFS worry scores range from 0 to 52; high scores indicate always engaging in behaviours to avoid low blood sugar or always worrying about concerns related to low blood sugar

There were 178 adverse events, including serious adverse events, experienced by 85 participants (52% for each group). Nine serious adverse events were reported for eight participants (four in each group), none related to the study device or procedure (Table 4).

**Table 4:** Adverse events

Variable	Intervention (*n* = 82)	Control (*n* = 81)
Participants with AEs or SAEs, *n* (%)	43 (52)	42 (52)
Total number of AEs or SAEs	92	86
Participants with SAEs, *n* (%)	4 (5)	4 (5)
Total number of SAEs	4	5
Participants with hypoglycaemic SAEs, *n* (%)^a^	1 (1)	3 (4)
Total number of hypoglycaemic SAEs^a^	1	4
Participants with hypoglycaemic AEs, *n* (%)	0	1 (1)
Total number of hypoglycaemic AEs	0	2
Participants with device-related AEs, *n* (%)^b^	6 (7)	0
Total number of device-related AEs	8	0
Participants discontinuing owing to AEs, *n* (%)	4 (5)	1 (1)^c^

Table includes the full analysis set and two participants who became pregnant

^a^A hypoglycaemic serious adverse event was reported during the baseline phase

^b^Device-related adverse events were all related to wearing the sensor: one participant with allergy (moderate), one with itching (mild), one with rash (mild), two with insertion-site symptom (four severe; one participant had three events and one participant had one event) and one with erythema (severe). Four intervention group participants withdrew owing to adverse events (two severe, two mild), which were primarily itching, redness and erythema

^c^Owing to severe hypoglycaemia

AE, adverse event; SAE, serious adverse event

Five hypoglycaemia-related serious adverse events were reported for four participants; one in the intervention group (one participant) and four in the control group (three participants). One control participant discontinued the study because of severe hypoglycaemia. In addition, one participant in the control group experienced two hypoglycaemia-related adverse events. There were no diabetic ketoacidosis events reported.

Eight adverse events for six (7%) intervention participants were related to wearing the study device. Four participants withdrew because of these adverse events (Table 4).

There were 144 sensor insertion-site symptoms experienced by 34 participants. The numbers of participants affected by expected signs or symptoms due to sensor insertion were: pain, *n* = 14; bleeding, *n* = 9; oedema, *n* = 3; and induration, *n* = 3. The symptoms associated with sensor wear were erythema, *n* = 23; itching, *n* = 14; and rash *n* = 8.

## Discussion

IMPACT was the first randomised controlled trial to assess the effect of flash sensor-based glucose monitoring on hypoglycaemia in adults with well-controlled type 1 diabetes as a replacement for SMBG [13]. The results from the original study are further supported by our findings of markedly reduced time and number of hypoglycaemic events in this pre-specified subgroup analysis of MDI-treated participants. To our knowledge, our study is the first to assess sensor-based monitoring in type 1 diabetes managed by MDI therapy with non-severe hypoglycaemia [21] as the primary endpoint. At present, there are limited data for CGM use in type 1 diabetes managed with MDI, the most common insulin therapy used in clinical practice [22]. Studies assessing the impact of CGM generally have HbA_1c_ as the primary endpoint for participants with suboptimally controlled type 1 diabetes; two recent studies in this cohort using MDI have added to this body of data [11, 12].

The pursuit of strict glycaemic control with analogue insulin has not fully realised the anticipated improved rates of hypoglycaemia in type 1 diabetes [23]. Frequent events of non-severe (self-treated) hypoglycaemia may be expected [24], particularly when glycaemic control is optimised [25]. A recent large global study suggests self-reported rates of mild hypoglycaemia are increasing [3]. These events may also be under-reported and missed by intermittent SMBG testing [26], rendering accurate clinical assessment of this issue challenging.

Clinically, a minimum reduction in hypoglycaemia of 30% is deemed significant [27] and glucose levels below the 3 mmol/l threshold should be considered serious and important hypoglycaemic events [28]. To date, use of standard CGM has shown reductions in time in hypoglycaemia (sensor glucose <3.9 mmol/l) [29] but usually in mixed insulin therapy cohorts [5, 6, 8, 9]. Recently, Beck et al showed improvement for time in hypoglycaemia over 24 h for MDI users and findings from the same study indicated a reduction in frequency of daily hypoglycaemic events [30].

Our findings of decreased time spent in hypoglycaemia <3.9 mmol/l equates to a 46% decrease in time compared with control participants, further improving to 58% less time at glucose levels <3.1 mmol/l and 63% at <2.5 mmol/l, and a corresponding, more marked, relative reduction in the number of events at lower hypoglycaemic thresholds. It has been speculated that this trend towards greater reductions in hypoglycaemia at lower thresholds might result from participants acting to treat hypoglycaemia only at a self-defined glucose level below 3.9 mmol/l [31], which may also be apposite to our findings.

Nocturnal hypoglycaemia remains a primary concern for individuals with type 1 diabetes [32] and was reported in the DCCT [1], but in few CGM studies since. Notably, our findings of significantly reduced exposure to hypoglycaemia were similarly observed at night (23:00–06:00 hours) at all lower glucose thresholds. These findings for significant reductions in overall and nocturnal hypoglycaemia lend further support to previous findings for improved hypoglycaemia with flash technology utilisation in diabetes treated with intensive insulin therapy [13, 33]. Similarly, the observed improvement in hypoglycaemia did not cause an analogous deterioration in overall glucose control, and HbA_1c_ measures remained essentially unchanged between the two groups. Studies in standard CGM use with insulin pump therapy indicate the largest glycaemic benefit is seen in those with a higher HbA_1c_ level and continued sensor wear [29]. In the two recent studies in type 1 diabetes and MDI therapy using standard CGM noted above, inclusion was restricted to participants with suboptimally controlled diabetes [11, 12]. Also, participants at increased risk of reduced concordance in using CGM were excluded.

The pivotal JDRF study demonstrated no benefit from CGM use in participants 14–24 years of age as only 30% of this group wore a sensor for more than 6 days per week [8]. Beck et al restricted participant inclusion to those over 25 years of age [12]. Post-randomisation, Lind et al excluded any participant unwilling to wear the device for more than 80% of the time [11]; sensor utilisation of 80% or more is considered necessary for meaningful benefit from continuous monitoring [29]. Moreover, both these studies incorporated diabetes education for participants, including interpretation of CGM data. In comparison, inclusion in our study was open to adults with type 1 diabetes of all ages (≥18 years) with an HbA_1c_ level ≤ 58 mmol/mol (7.5%), making any improvement in glycaemic control less certain. Prior to randomisation, during the masked-mode baseline phase, the minimum threshold of acquiring approximately 50% (650 historic results) sensor glucose data was to ensure adequate data for analysis. Participants in our study did not receive face-to-face training for the system or any other protocol-stipulated insulin dose education. This pre-specified subgroup of MDI therapy users could be considered diabetes-technology naive as they were neither CGM nor insulin pump users. Nevertheless, ease of use of the system and fast adaptation to sensor glucose data are verified by the marked reductions in hypoglycaemia, a considerable number of which coincided immediately with unmasking of the sensor and initial use of sensor glucose results by the participants and prior to a review with a clinician. Participant utilisation of the device was high, at over 92%, and maintained throughout the study. General acceptance of the system to support self-management was also demonstrated by the improvements in diabetes quality of life satisfaction with treatment score and the overall diabetes treatment satisfaction score. Likewise, improvement in perceived awareness of hypo- and hyperglycaemia suggests confidence in sensor glucose results.

The change in total daily dose of insulin was similar in both groups at the end of the study. However, the significant decrease in hypoglycaemia observed in the intervention group cannot be explained by behaviour modifications alone as these will usually only cause a small decrease in hypoglycaemia [29]. A similar pattern of insulin use was observed in our primary study of MDI and insulin pump use [13], with flash technology in type 2 diabetes managed with MDI [33] and with standard CGM in type 1 diabetes managed by MDI or insulin pump therapy [34]. As an insulin algorithm or treatment protocol were not included in our study and participants did not have the flexibility and sensitivity/responsiveness of insulin pump therapy, we speculate the use of sensor glucose data prompted minor daily modifications to insulin dose or administration time which did not impact total doses or proportions of insulin overall. The dramatic decrease in blood glucose testing frequency supports the replacement use of sensor glucose data to promote these changes to insulin administration. Additionally, a recent small study assessing use of glucose trend arrows with standard CGM observed that individuals with type 1 diabetes often rely on this additional information to adjust insulin doses [35].

Adverse events for skin reactions occurred in 7% of participants which was similar to our observations in the IMPACT trial (8%) [13], for type 2 diabetes managed with MDI [33], and is as expected for medical-grade adhesive use to attach a device to the body.

Our study adds to the increasing number of sensor-based glucose monitoring studies conducted in type 1 diabetes managed with MDI and those using flash technology. However, there are limitations which affect the general applicability of the results. Many of the endpoints, particularly those derived from sensor glucose values, are highly inter-related and should not be considered in isolation, as no adjustment was made for multiple testing of secondary endpoints. This pre-specified subgroup was limited to adults with well-controlled type 1 diabetes managed with MDI therapy, which may suggest these participants were more motivated or committed to self-management than other MDI populations. Future studies could consider broader inclusion criteria, including suboptimal glycaemic control and younger age participants with diverse race or ethnicity, to reflect the real-world population with diabetes. In addition, a crossover study design may add to the evidence that continuous monitoring has an effect only concurrent with treatment [10, 31], and a longer duration study may demonstrate sustained avoidance of hypoglycaemia with continued use of the device in type 1 diabetes, similar to use in type 2 diabetes [33]. As noted in our primary study and another recent study in MDI users, neither participants nor investigators were blinded to the intervention, which may have influenced the treatment effect [28].

The strength of our findings is in demonstrating the glycaemic control benefit from use of flash sensor technology by MDI therapy users with type 1 diabetes, indicating the improvement is associated with the information provided by the technology not a specific insulin treatment modality. This is similar to findings for standard CGM use and impaired awareness of hypoglycaemia [31].

To summarise, use of the flash glucose sensor system in participants with well-controlled type 1 diabetes managed with MDI resulted in significant reductions in time and frequency of hypoglycaemia, with no change in HbA_1c_ levels. Safety of the system as a replacement for SMBG has been demonstrated, together with high acceptance of the system by participants. Use of this sensor-based system may contribute to effective management for optimal glucose control, which is currently constrained by hypoglycaemia for many individuals with type 1 diabetes.

## Electronic supplementary material

ESM (PDF 108 kb)

## Abbreviations

CGM: Continuous glucose monitoring
CSII: Continuous subcutaneous insulin infusion
DDS: Diabetes Distress Scale
DQoL: Diabetes Quality of Life Questionnaire
DTSQ: Diabetes Treatment Satisfaction Questionnaire
HFS: Hypoglycaemia Fear Survey
IMPACT: Novel glucose-sensing technology and hypoglycemia in type 1 diabetes: a multicentre, non-masked, randomised controlled trial
MDI: Multiple daily injection
SMBG: Self-monitoring of blood glucose

## References

[1] DCCT Research Group (1991). Epidemiology of severe hypoglycemia in the diabetes control and complications trial. Am J Med.

[2] Nathan DM, Cleary PA, Backlund JY (2005). Diabetes Control and Complications Trial/Epidemiology of Diabetes Interventions and Complications (DCCT/EDIC) Study Research Group. Intensive diabetes treatment and cardiovascular disease in patients with type 1 diabetes. N Engl J Med.

[3] Khunti K, Alsifri S, Aronson R, the HAT Investigator Group (2016). Rates and predictors of hypoglycemia in 27585 people from 24 countries with insulin-treated type 1 and type 2 diabetes: the global HAT study. Diabetes Obes Metab.

[4] Cryer PE (2004). Diverse cause of hypoglycaemia-associated autonomic failure in diabetes. N Engl J Med.

[5] Juvenile Diabetes Research Foundation Continuous Glucose Monitoring Study Group (2009). The effect of continuous glucose monitoring in well-controlled type 1 diabetes. Diabetes Care.

[6] Battelino T, Phillip M, Bratina N, Nimri R, Oskarsson P, Bolinder J (2011). Effect of continuous glucose monitoring on hypoglycemia in type 1 diabetes. Diabetes Care.

[7] Deiss D, Bolinder J, Riveline JP (2006). Improved glycemic control in poorly controlled patients with type 1 diabetes using real-time continuous glucose monitoring. Diabetes Care.

[8] The Juvenile Diabetes Research Foundation Continuous Glucose Monitoring Study Group (2008). Continuous glucose monitoring and intensive treatment of type 1 diabetes. N Engl J Med.

[9] Šoupal J, Petruželková L, Flekač M (2016). Comparison of different treatment modalities for type 1 diabetes, including sensor-augmented insulin regimens, in 52 weeks of follow-up: a COMISAIR study. Diabetes Technol Ther.

[10] Battelino T, Conget I, Olsen B (2012). The use and efficacy of continuous glucose monitoring in type 1 diabetes treated with insulin pump therapy: a randomised controlled trial. Diabetologia.

[11] Lind M, Polonsky W, Hirsch IB (2017). Continuous glucose monitoring vs conventional therapy for glycemic control in adults with type 1 diabetes treated with multiple daily insulin injections. The GOLD Randomized Clinical Trial. JAMA.

[12] Beck RW, Riddlesworth T, Ruedy K (2017). Effect of continuous glucose monitoring on glycemic control in adults with type 1 diabetes using insulin injections the DIAMOND Randomized Clinical Trial. JAMA.

[13] Bolinder J, Antuna R, Geelhoed-Duijvestijn P, Kröger J, Weitgasser R (2016). Novel glucose-sensing technology and hypoglycemia in type 1 diabetes: a multicentre, non-masked, randomised controlled trial. Lancet.

[14] Bailey T, Bode BW, Christiansen MP (2015). The performance and usability of a factory-calibrated flash glucose monitoring system. Diabetes Technol Ther.

[15] Abbott Diabetes Care FreeStyle Libre Software. Available from www.FreeStyleLibre.co.uk. Accessed 23 Nov 2017

[16] Kovatchev BP, Clarke WL, Breton M, Brayman K, McCall A (2005). Quantifying temporal glucose variability in diabetes via continuous glucose monitoring: mathematical methods and clinical application. Diabetes Technol Ther.

[17] Polonsky WH, Fisher L, Earles J (2005). Assessing psychosocial distress in diabetes: development of the Diabetes Distress Scale. Diabetes Care.

[18] The DCCT Research Group (1988). Reliability and validity of a diabetes quality-of-life measure for the diabetes control and complications trial (DCCT). Diabetes Care.

[19] Bradley C (1994) Diabetes treatment satisfaction questionnaire. In: Bradley C (ed) Handbook of psychology and diabetes. Harwood Academic Publishers, pp 111–132

[20] Gonder-Frederick LA, Schmidt KM, Vajda KA (2011). Psychometric properties of the hypoglycemia fear survey-II for adults with type 1 diabetes. Diabetes Care.

[21] Workgroup on Hypoglycaemia, American Diabetes Association (2005). Defining and reporting hypoglycaemia in diabetes: a report from the American Diabetes Association Workgroup on Hypoglycaemia. Diabetes Care.

[22] Pozzilli P, Battelino T, Danne T, Hovorka R, Jarosz-Chobot P, Renard E (2016). Continuous subcutaneous insulin infusion in diabetes: patient populations, safety, efficacy, and pharmacoeconomics. Diabetes Metab Res Rev.

[23] Kristensen PL, Hansen LS, Jespersen MJ (2012). Insulin analogues and severe hypoglycaemia in type 1 diabetes. Diabetes Res Clin Pract.

[24] Frier BM (2014). Hypoglycaemia in diabetes mellitus: epidemiology and clinical implications. Nat Rev Endocrinol.

[25] McCrimmon RJ, Sherwin RS (2010). Hypoglycemia in type 1 diabetes. Diabetes.

[26] Toschi E, Wolpert H (2016). Utility of continuous glucose monitoring in type 1 and type 2 diabetes. Endocrinol Metab Clin.

[27] Seaquist ER, Anderson J, Childs B (2013). Hypoglycemia and diabetes: a report of a workgroup of the American Diabetes Association and the Endocrine Society. Diabetes Care.

[28] The International Hypoglycaemia Study Group (2017). Glucose concentrations of less than 3.0 mmol/l (54 mg/dl) should be reported in clinical trials: a joint position statement of the American Diabetes Association and the European Association for the Study of Diabetes. Diabetologia.

[29] Pickup JC, Freeman SC, Sutton AJ (2011). Glycaemic control in type 1 diabetes during real time continuous glucose monitoring compared with self-monitoring of blood glucose: meta-analysis of randomised controlled trials using individual patient data. BMJ.

[30] Riddlesworth T, Price D, Cohen N, Beck RW (2017). Hypoglycemic event frequency and the effect of continuous glucose monitoring in adults with type 1 diabetes using multiple daily injections. Diabetes Ther.

[31] van Beers CA, DeVries JH, Kleijer SJ (2016). Continuous glucose monitoring for patients with type 1 diabetes and impaired awareness of hypoglycaemia (IN CONTROL): a randomised, open-label, crossover trial. Lancet Diabetes Endocrinol.

[32] van Beers CA, DeVries JH (2016). Continuous glucose monitoring: impact on hypoglycaemia. J Diabetes Sci Technol.

[33] Haak T, Hanaire H, Ajjan R, Normanns H, Rayman G (2017). Flash glucose-sensing technology as a replacement for blood glucose monitoring for the management of insulin-treated type 2 diabetes: a multicenter, open-label randomized controlled trial. Diabetes Ther.

[34] Riveline J-P, Schaepelynck P, Chaillous L, for the EVADIAC Sensor Study Group (2012). Assessment of patient led or physician-driven continuous glucose monitoring in patients with poorly controlled type 1 diabetes using basal bolus regimens. A 1-year multicenter study. Diabetes Care.

[35] Pettus J, Edelman S (2016). Use of glucose rate of change arrows to adjust insulin therapy among individuals with type 1 diabetes who use continuous glucose monitoring. J Diabetes Sci Technol.

