# Supplementary material for: Impact of flash glucose monitoring on hypoglycaemia in adults with type 1 diabetes managed with multiple daily injection therapy: a pre-specified subgroup analysis of the IMPACT randomised controlled trial
Source: Diabetologia. 2017 Dec 23;61(3):539–50. doi: 10.1007/s00125-017-4527-5 (PMC6448969; doi:10.1007/s00125-017-4527-5)
Supplement: Supplementary file 1 — (PDF 108 kb) [file 125_2017_4527_MOESM1_ESM.pdf]

Electronic supplementary material (ESM) for:

Impact of flash glucose monitoring on hypoglycaemia in adults with type 1 diabetes managed with multiple daily injection therapy: a pre-specified subgroup analysis of the IMPACT randomised controlled trial

Per Oskarsson, Ramiro Antuna, Petronella Geelhoed-Duijvestijn, Jens Kröger, Raimund Weitgasser

Jan Bolinder

ESM table 1: Mean number of glucose readings used in the primary endpoint analysis

	**Baseline**		**Study End**	
	**Intervention (n=81)**	**Control (n=79)**	**Intervention (n=81)**	**Control (n=79)**
Overall 24 h	1265	1269	1364	1172
Daytime	901	902	968	827
Night-time	364	367	396	345

ESM table 2: Data used for the primary analysis where 72 h of data in the final 2 weeks (days 194–208) were not available

**Number of participants**	**Baseline**	**Days 45–75**	**Days 75–91**	**Days 91–105**	**Days 105–194**	**Days 105–208**
Intervention[a]	0	2	1	1	2	2
Control	10			1		

[a]Two intervention arm participants had some data in days 194–208 but less than 72 h, so data from the period prior to day 194 were added to make up the 72 h

ESM table 3: Glycaemic and glucose variability measures at 3 months

Variable	Baseline		Study end		Difference in adjusted means between intervention and control (95% CI)	Difference in intervention vs control (%)	*p* value
	Intervention (n=81)	Control (n=79)	Intervention (n=81)	Control (n=79)			
HbA_{1c} (mmol/mol)	50.8 (4.8)	49.9 (7.5)	52.2 (6.3)	51.6 (7.5)	-0.1 (-1.7, 1.5)	NA	0.91
HbA_{1c} (%)	6.80 (0.44)	6.71 (0.69)	6.92 (0.58)	6.87 (0.68)	-0.01 (-0.15, 0.14)	NA	0.91
Time in glucose 3.9–10.0 mmol/l ((h)	15.0 (2.6)	14.3 (2.9)	15.8 (2.8)	13.8 (3.0)	1.6 (0.9, 2.3)	11.4	<0.0001
**Glucose <3.9 mmol/l**							
24 h period							
Events	1.80 (0.80)	1.72 (0.75)	1.25 (0.68)	1.65 (0.84)	-0.45 (-0.65, -0.24)	-26.6	<0.0001
Duration (h)	3.44 (2.10)	3.73 (2.72)	1.88 (1.32)	3.39 (2.37)	-1.37 (-1.84, -0.90)	-41.2	<0.0001
AUC (h x mmol/l)	3.17 (2.57)	3.60 (3.38)	1.46 (1.35)	3.20 (2.93)	-1.56 (-2.15, -0.97)	-50.1	<0.0001
Night period (23:00 – 06:00 hours)							
Events	0.57 (0.34)	0.61 (0.38)	0.35 (0.27)	0.55 (0.33)	-0.19 (-0.27, -0.11)	-35.1	<0.0001
Time in h	1.20 (0.89)	1.41 (1.12)	0.66 (0.65)	1.33 (0.95)	-0.59 (-0.81, -0.37)	-45.7	<0.0001
**Glucose <3.1 mmol/l**							
24 h period							
Events	1.01 (0.65)	1.00 (0.69)	0.52 (0.44)	0.90 (0.68)	-0.39 (-0.54, -0.25)	-43.5	<0.0001
Duration (h)	1.75 (1.53)	1.99 (1.97)	0.75 (0.81)	1.76 (1.73)	-0.90 (-1.25, -0.55)	-52.9	<0.0001
AUC (h x mmol/l)	1.00 (1.07)	1.20 (1.39)	0.39 (0.48)	1.06 (1.25)	-0.60 (-0.86, -0.34)	-58.7	<0.0001
Night period (23:00 – 06:00 hours)							
Events	0.37 (0.27)	0.41 (0.34)	0.17 (0.19)	0.35 (0.24)	-0.16 (-0.22, -0.10)	-47.2	<0.0001
Duration (h)	0.67 (0.62)	0.85 (0.85)	0.31 (0.42)	0.78 (0.73)	-0.40 (-0.57, -0.23)	-53.7	<0.0001
**Glucose <2.5 mmol/l** [a]							
24 h period							
Events	0.61 (0.55)	0.63 (0.59)	0.26 (0.34)	0.59 (0.63)	-0.32 (-0.45, -0.19)	-54.1	<0.0001
Duration (h)	0.97 (1.15)	1.19 (1.48)	0.35 (0.49)	1.05 (1.37)	-0.62 (-0.91, -0.34)	-61.4	<0.0001
AUC (h x mmol/l)	0.26 (0.34)	0.32 (0.44)	0.09 (0.13)	0.28 (0.40)	-0.17 (-0.26, -0.09)	-63.6	<0.0001
Night period (23:00 – 06:00 hours)							
Events	0.26 (0.25)	0.30 (0.32)	0.10 (0.15)	0.24 (0.23)	-0.12 (-0.18, -0.07)	-53.1	<0.0001
Duration (h)	0.40 (0.46)	0.56 (0.69)	0.16 (0.26)	0.51 (0.62)	-0.30 (-0.44, -0.16)	-61.6	<0.0001

Variable	Baseline		Study end		Difference in adjusted means between intervention and control (95% CI)	Difference in intervention vs control (%)	*p* value
	Intervention (n=81)	Control (n=79)	Intervention (n=81)	Control (n=79)			
**Glucose <2.2 mmol/l**							
24 h period							
Events	0.44 (0.48)	0.49 (0.52)	0.17 (0.25)	0.44 (0.56)	-0.25 (-0.37, -0.13)	-58.0	<0.0001
Duration (h)	0.69 (0.97)	0.88 (1.24)	0.23 (0.36)	0.77 (1.18)	-0.48 (-0.73, -0.23)	-64.6	0.0002
**Duration (h) at hyperglycaemic glucose level within 24 h period**							
>10.0 mmol/l	5.6 (2.4)	6.0 (3.3)	6.3 (2.9)	6.8 (3.6)	-0.3 (-1.1, 0.5)	-4.2	0.50
>13.3 mmol/l	1.77 (1.36)	2.05 (1.86)	1.73 (1.37)	2.50 (2.15)	-0.60 (-1.06, -0.14)	-24.8	0.011
>16.7 mmol/l	0.44 (0.50)	0.57 (0.77)	0.32 (0.37)	0.63 (0.91)	-0.26 (-0.46, -0.06)	-43.3	0.011
**Glucose variability**							
BGRI	8.1 (2.3)	8.7 (2.9)	7.4 (2.4)	9.2 (3.1)	-1.4 (-2.1, -0.7)	-16.0	<0.0001
CV glucose (%)	43.2 (6.6)	43.4 (6.5)	37.9 (6.2)	42.3 (6.6)	-4.3 (-5.8, -2.8)	-10.2	<0.0001
LBGI	2.7 (1.4)	2.9 (1.8)	1.7 (1.0)	2.6 (1.5)	-0.8 (-1.1, -0.5)	-31.9	<0.0001
MAGE (mmol/l)	7.9 (1.5)	8.2 (1.8)	7.5 (1.4)	8.3 (1.9)	-0.63 (-1.04, -0.22)	-7.8	0.0026
Mean glucose (mmol/l)	7.8 (1.0)	7.9 (1.4)	8.2 (1.0)	8.2 (1.5)	0.01 (-0.30, 0.32)	0.1	0.94
SD of glucose (mmol/l)	3.4 (0.6)	3.4 (0.8)	3.1 (0.6)	3.5 (0.8)	-0.35 (-0.51, -0.19)	-10.1	<0.0001
CONGA 2h (mmol/l)	3.2 (0.7)	3.2 (0.8)	2.8 (0.7)	3.4 (0.8)	-0.60 (-0.79, -0.42)	-17.9	<0.0001
CONGA 6h (mmol/l)	4.0 (1.3)	4.0 (1.5)	3.2 (1.2)	4.1 (1.6)	-0.87 (-1.29, -0.45)	-21.4	<0.0001

Data are mean (SD) unless otherwise stated

[a]Post hoc endpoint

BGRI, blood glucose risk index; CONGA, continuous overall net glycaemic action; LBGI, low blood glucose index; MAGE, mean amplitude of glycaemic excursions

Baseline sensor data was not available for analysis for one control participant.

List of study centres

23 study centres (three in Sweden, six in Austria, five in Germany, three in Spain, and six in the Netherlands).
Chief Investigator (Sweden): J Bolinder, Karolinska Universitetssjukhuset Huddinge, Stockholm
Other Swedish investigators:
M Alvarsson, Karolinska Universitetssjukhuset Solna, Stockholm
P Hallgren, Falu Lasarett, Falun

Country Lead Principal Investigator (Austria): R Weitgasser, Diakonissen-Krankenhaus Salzburg, Abteilung für Innere Medizin, Salzburg
Other Austrian investigators:
T Pieber, Medizinische Universität Graz, Graz
P Fasching, Wilhelminenspital Medizin, Wien
H Drexel, VIVIT-Institut am Akad. Lehrkrankenhaus Feldkirch, Feldkirch
A Luger, Medizinische Universität Wien, Wien
M Laimer/Dr Christoph Ebenbichler, Universitätsklinik für Innere Medizin I, Innsbruck

Country Lead Principal Investigator (Germany): S Matthaei, Christliches Krankenhaus Quakenbrück gemeinnützige GmbH, Quakenbrück
Other German investigators:
R Geldmacher, Diabetes-Zentrum Hannover-Nord, Hannover
J Kröger, Zentrum für Diabetologie Hamburg-Bergedorf, Hamburg
T Haak, Diabetes Zentrum Mergentheim, Bad Mergentheim
U Aigner, Versdias GmbH, Sulzbach-Rosenberg

Country Lead Principal Investigator (Spain): R Antuna, Clinica Diabetologica, Gijon
Other Spanish investigators:
E Aguilera, Hospital Universari Germans Trias i Pujol, Badalona
S Gaztambide Sáenz, Servicio de Endocrinología Hospital Unversitario Cruces, Cruces-Barakaldo (Vizcaya)

Country Lead Principal Investigator (Netherlands): P Geelhoed, Medisch Centrum Haaglanden, Den Haag
Other Dutch investigators:
S van Thiel, Amphia Ziekenhuis, Breda
F Storms, St. Antonius, Utrecht
T Brouwer, Onze Lieve Vrouwe Gasthuis (OLVG), Amsterdam
E Serne, VU University Medical Center, Amsterdam
A Kooy, Bethesda Diabetes Research Center, Hoogeveen
